# Antimicrobial Resistance (AMR) Surveillance: Ten Years On

**DOI:** 10.1093/cid/ciag496

**Published:** 2026-10-19

**Authors:** Dame Sally C Davies

**Affiliations:** Trinity College, University of Cambridge, Cambridge, United Kingdom

Antimicrobial resistance threatens to reverse decades of medical progress, turning once-routine infections into life-threatening conditions and placing growing strain on health systems and economies worldwide. The urgency to act has never been greater: without decisive intervention, an estimated 39 million deaths could be attributable to AMR between 2025 and 2050 [1], while the global economy could be up to US$1.7 trillion smaller annually by mid-century [2].

While the immediate impacts are often most visible in resource-constrained settings, the drivers and consequences of AMR are inherently global—affecting all countries and underscoring the need for collective, system-wide solutions.

The Fleming Fund was established in 2015 to address a central weakness in the global AMR response: the lack of reliable, cross-sectoral data in many low- and middle-income countries needed to inform national action and global efforts. Few international initiatives have combined investment in laboratory capacity, workforce development, governance, and One Health surveillance at this scale across multiple low- and middle-income countries. In doing so, the Fund has generated lessons that extend well beyond its partner countries, offering important insights for strengthening surveillance systems that underpin global health security, outbreak detection, and pandemic preparedness.

Over nearly a decade, the Fund successfully supported countries in Africa and Asia to strengthen AMR surveillance through investment in laboratories, workforce development, governance, and data systems, guided by a One Health perspective. It expanded bacteriology and antimicrobial susceptibility testing, strengthened reference laboratories, and supported engagement with global surveillance platforms, including WHO's Global Antimicrobial Resistance and Use Surveillance System (GLASS). These efforts demonstrate how sustained investment in surveillance capacity can enhance not only AMR response but also the broader ability of health systems to detect, interpret, and respond to emerging threats.

The Fleming Fund formally closed in early 2026, marking a transition rather than completion of the AMR agenda. While much has been achieved, too many surveillance systems remain dependent on external financing, the processes needed to routinely translate surveillance data into decisions are still developing, and long-term sustainability gains cannot be assumed—particularly amid constrained global financing and growing pressure on health systems.

This journal contributes to the Fleming Fund's legacy, synthesizing learning from the program implementation and evaluation to inform future AMR surveillance and action. Drawing on evaluation evidence, country experience, and practitioner reflection, six papers address the core elements of a functional surveillance system.

1. The first paper sets out the Fund's scope, governance, and operating model.
2. The second paper examines how the Fund strengthened AMR data generation, focusing on the systems-based approach linking laboratories, quality assurance, workforce development, and data platforms.
3. The third paper explores how AMR data have been used to inform action across facility, sub-national, national, and regional levels, showing that leadership, incentives, and institutional demand matter as much as data quality.
4. The fourth paper reflects on experience with One Health approaches, illustrating variations in multisectoral governance and why integration proved difficult in practice.
5. The fifth paper presents a country-level case study from Kenya, tracing how sustained investment strengthened One Health governance, expanded surveillance, and increased use of AMR data within a devolved system.
6. The final paper considers the future of AMR surveillance and action, asking what it will take for systems to remain functional, affordable, and action-oriented in a changing global landscape.

Together, these papers underline how AMR surveillance is a long-term public good. Building it requires realism about costs and constraints; using it effectively requires clarity of purpose, continued investment in people and institutions, and sustained political commitment.

The lessons captured in this series are therefore relevant not only to AMR but to the wider global effort to build resilient, data-driven systems capable of addressing current and future health threats.

This series is offered as a foundation for future action.
